# Oocyte Meiotic Competence in the Domestic Cat Model: Novel Roles for Nuclear Proteins BRD2 and NPM1

**DOI:** 10.3389/fcell.2021.670021

**Published:** 2021-05-03

**Authors:** Daniela R. Chavez, Pei-Chih Lee, Pierre Comizzoli

**Affiliations:** Smithsonian Conservation Biology Institute, National Zoological Park, Washington, DC, United States

**Keywords:** oocyte, meiotic competence, domestic cat, fertility, germinal vesicle

## Abstract

To participate in fertilization and embryo development, oocytes stored within the mammalian female ovary must resume meiosis as they are arrested in meiotic prophase I. This ability to resume meiosis, known as meiotic competence, requires the tight regulation of cellular metabolism and chromatin configuration. Previously, we identified nuclear proteins associated with the transition from the pre-antral to the antral follicular stage, the time at which oocytes gain meiotic competence. In this study, the objective was to specifically investigate three candidate nuclear factors: bromodomain containing protein 2 (BRD2), nucleophosmin 1 (NPM1), and asparaginase-like 1 (ASRGL1). Although these three factors have been implicated with folliculogenesis or reproductive pathologies, their requirement during oocyte maturation is unproven in any system. Experiments were conducted using different stages of oocytes isolated from adult cat ovaries. The presence of candidate factors in developing oocytes was confirmed by immunostaining. While BRD2 and ASRGL1 protein increased between pre-antral and the antral stages, changes in NPM1 protein levels between stages were not observed. Using protein inhibition experiments, we found that most BRD2 or NPM1-inhibited oocytes were incapable of participating in fertilization or embryo development. Further exploration revealed that inhibition of BRD2 and NPM-1 in cumulus-oocyte-complexes prevented oocytes from maturing to the metaphase II stage. Rather, they remained at the germinal vesicle stage or arrested shortly after meiotic resumption. We therefore have identified novel factors playing critical roles in domestic cat oocyte meiotic competence. The identification of these factors will contribute to improvement of domestic cat assisted reproduction and could serve as biomarkers of meiotically competent oocytes in other species.

## Introduction

Mammalian reproduction relies on oocytes that develop the exclusive capacity to participate in fertilization and support early embryo development. The pool of early stage oocytes within the female ovary suspend development at birth, and remain in meiotic prophase I, also called the germinal vesicle (GV) stage. At this stage, oocytes are incapable of fertilization until hormonal cyclicity begins, when GV oocytes can resume meiosis and mature before fertilization. The ability to exit meiotic prophase I and resume meiosis is known as meiotic competence, which is gained when oocytes develop from the immature pre-antral stage to the antral stage. During this transition, oocytes grow in size, and store critical proteins and nutrients needed for meiotic resumption, all of which is critical to sexual reproduction (Coticchio et al., 2015).

Important cellular maturation events occur in the nucleus and cytoplasm of oocytes during meiotic maturation. These events include chromatin condensation during germinal vesicle breakdown (GVBD), chromatin modifications and remodeling, chromosome segregation mediated by the meiotic spindle, polar body extrusion, changes to the cytoplasmic metabolic environment and cell-cell signaling with adjacent somatic cells. Each of these events is critical to sustain such a large cell through fertilization and early embryo development (Coticchio et al., 2015; Clarke, 2018; Conti and Franciosi, 2018). Importantly, the GV oocyte accomplishes these processes almost exclusively without new transcription and relies on stored RNA to generate the proteins required for these processes. Thus, the GV oocyte is fully loaded with the cellular machinery and proteins required to perform these dramatic changes.

To increase our knowledge of oocyte development, our laboratory, as well as other research teams, have used the domestic cat as a model. The domestic cat is a critical biomedical model to complement and expand findings in rodent models as several aspects of feline reproduction more accurately represent human reproductive biology than do rodent models. For example, oocyte size, asymmetrical embryonic divisions, a shared environment with humans and a genome with extensive synteny to the human genome make the cat a cost-effective, non-invasive biomedical model that could improve artificial reproductive technology (ART) in humans (O’Brien et al., 2001). It also is an excellent model for rare and endangered felids (O’Brien et al., 2001; Herrick, 2019; Thongphakdee et al., 2020). Specifically, understanding oocyte maturation is essential to develop fertility treatments for both humans and endangered felids. Both can greatly benefit from improved *in vitro* maturation (IVM) of GV oocytes as they can be acquired without what is often harmful hormonal stimulation (Thongphakdee et al., 2020).

In the cat model, GVs acquire full meiotic competence during the transition from the pre-antral to the antral stage. This has been demonstrated using GV transfer experiments showing that antral, but not pre-antral GVs transferred to mature cytoplasts are capable of meiotic maturation (Comizzoli et al., 2011). In a recent proteomics study, the levels of 74 GV proteins were found to change during the transition from the pre-antral to antral stage (Lee et al., 2018). Our aim in the current study was to validate and further characterize candidate proteins that can serve as markers of competent oocytes. Identification of these markers could help expand our understanding of oocyte development and be applied to improve ART by using them to assess the efficacy of female fertility preservation methods, especially ones focusing on preservation of the GV alone (Graves-Herring et al., 2013; Elliott et al., 2015; Lee and Comizzoli, 2019).

Selection of proteins to investigate was based on the following criteria: (1) proteins with various functions known to be critical to oocyte development such as chromatin structure/regulation and cellular metabolism, (2) proteins previously associated with reproduction/oocytes, and (3) proteins not extensively studied in oocytes yet. Thus, we chose three proteins; bromo-domain-containing 2 (BRD2), nucleophosmin 1 (NPM1), and asparaginase like-1 (ASRGL1). BRD2 (also known as female sterile homeotic related gene-1, Fsrg1) is a bromodomain extra terminal protein that is broadly expressed in many cell types including reproductive tissues such as the ovary, oocytes, uterus, and testis (Rhee et al., 1998; Trousdale and Wolgemuth, 2004; Smith and Zhou, 2016). BRD2 is known to regulate transcription, is important for DNA double-stranded break repair and is critical for embryonic development as null BRD2 mutant mice display embryonic lethality with neural tube defects (Gyuris et al., 2009; Hnilicová et al., 2013; Gursoy-Yuzugullu et al., 2017). While expression of BRD2 has been observed throughout mouse folliculogensis, it has not been tested for a role in the oocyte (Rhee et al., 1998; Trousdale and Wolgemuth, 2004; Smith and Zhou, 2016; Mathur et al., 2017; Lim et al., 2019). NPM1 is a histone chaperone important in ribosome biogenesis, regulation of apoptosis and chromatin remodeling (Box et al., 2016; Yu et al., 2021). Additionally, misregulation of NPM1 has been implicated in various cancers (Lim et al., 2019; Kuravi et al., 2021; Yu et al., 2021). However, a role for NPM1 during folliculogenesis has not been identified. Finally, we selected a protein important for cellular metabolism. ASRGL1 is a ubiquitously-expressed aspariginase enzyme present in the mammalian uterus and is highly expressed in the brain and testes (Bush et al., 2002; Dieterich et al., 2003). Recently, ASRGL1 has been explored as a biomarker for uterine carcinomas (Edqvist et al., 2015; Huvila et al., 2018). To date, a role of ASRGL1 in the oocyte has not been identified.

The objective of the study was to identify novel factors that contribute to oocyte meiotic competence using the domestic cat model. Specifically, our aim was to test the role of three candidate proteins that we previously identified to be associated with oocyte development during the transition from the less competent pre-antral to the meiotically competent antral stage. We hypothesized that changes in the protein levels in the nucleus observed previously by our group suggested these factors are potential contributors to overall fertility by regulating oocyte maturation and/or embryo development.

## Materials and Methods

### Ovarian Follicle and Oocyte Collection

Ovaries from adult (>1 year) domestic cats were obtained after routine ovariohysterectomy from local veterinary clinics. The study did not require the approval of the Animal Care and Use Committee of the Smithsonian Conservation Biology Institute because cat ovaries were collected at local veterinary clinics as byproducts from owner-requested routine ovariohysterectomies. Reproductive tracts were immediately stored in PBS (1X Gibco Dulbecco’s Phosphate-Buffered Saline, 0.5 mg/mL penicillin and streptomycin) at 4°C for transportation to the laboratory and used for experiments within 24 h of surgeries (Johnston et al., 1989; Wood et al., 1997; Otoi et al., 2001; Naoi et al., 2007). Pre-antral follicles (100–400 μm, and one layer of granulosa cells) and grade 1 and grade 2 (>400 μm, follicles with a fluid-filled antrum and > 3 layers of cumulus cells) antral stage cumulus cell-oocyte complexes (COCs) from antral follicles were mechanically isolated from ovaries into HEPES (Sigma)-buffered minimal essential medium (MEM, Gibco) supplemented with 100 μg/mL streptomycin, 100 μg/mL penicillin, 4 mg/mL bovine serum albumen (BSA), 2 mM L-glutamine, and 1 mM pyruvate (Wood and Wildt, 1997; Lee et al., 2018).

### Validation of Antibody Specificity for Candidate Proteins

To validate the specificity of the antibodies used, western blots were performed for each protein investigated using ovarian tissue lysate (Supplementary Figure 1). Half an ovary was snap frozen until used for western blots. Thawed tissue was repeatedly sliced on ice and cells lysed in RIPA buffer (R0278 [Sigma]) containing a mix of protease inhibitors [P8340 ([Sigma)] and 100 μM phenylmethylsulfonly fluoride. Tissue was sonicated on ice for 3 × 10 s at 60% amplitude. SDS sample buffer (Bio-Rad) was added and lysates were incubated for 95°C for 10 min and briefly maintained on ice prior to being resolved by SDS-PAGE [pre-made 4–15% Mini-Pro-TEAN TGX precast gels (Bio-Rad) or hand-made 10%] together with Precision Plus Protein Dual Color molecular weight standard (Bio-Rad). Proteins were transferred to activated polyvinylidene fluoride membranes (Millipore Sigma). Membranes were blocked for 1 h at room temperature in 4% BSA then incubated in primary antibodies overnight at 4°C. Following 3 × 10 min washes, membranes were incubated in secondary antibody [anti-mouse or rabbit IgG conjugated to horseradish peroxidase (Sigma)] and washed 3 × 10 min each. Chemiluminescence was detected with Clarity Western ECL Substrate (Bio-Rad, United States) and imaged with the PXi4 imaging system (Syngene).

### Immunostaining of Proteins (Experiment 1)

Oocytes from pre-antral (*n* = 55) and antral follicles (*n* = 66) were pooled from different ovaries (in at least 2 replicates) and allocated to one of the three protein staining groups. To visualize proteins within the GV, granulosa cells were mechanically removed from pre-antral oocytes by dissecting with two needles and removed from antral oocytes using a stripper pipette. Antibodies against BRD2 (Abcam 139690), NPM-1 (Abcam 10530), and ASRGL1 (Proteintech 11400-1-AP) were used for identification of protein localization. Oocytes were fixed in 4% paraformaldehyde (PFA) in PBS (Thermo Fisher Scientific) for 30 min at room temperature or at 4°C overnight. Fixed oocytes were rinsed twice in wash solution [2% fetal bovine serum (FBS, Irvine Scientific, United States), 0.5% Triton X-100 in PBS] and blocked twice in saturation solution (20% FBS, 0.5% triton X-100 in PBS) for 15 min at 38.5°C. Oocytes were incubated with primary antibodies (1:500 dilution for each) in saturation solution at 4°C overnight. As a negative control, non-immune IgG from rabbit or mouse was used on a subset of oocytes (Supplementary Figure 2). Following three 10 min washes, cells were incubated with a fluorescein isothiocyanate (FITC)-conjugated secondary antibody [anti-mouse F0257 or anti-rabbit F0382 (Sigma)] in wash solution at 38.5°C for 1 h followed by three washes at 38.5°C and mounted on slides with mounting medium [Vectashield with DAPI (Vector Laboratories)] containing DAPI. Stained oocytes were imaged using an Olympus BX41 epifluorescence microscope and SPOT software version 5.0.27. The excitation and detection wavelengths were 488 and 492–544 nm for FITC, respectively, and 405 and 415–487 nm for DAPI. Relative nuclear and cytoplasmic fluorescence intensity was quantified using ImageJ software (National Institutes of Health, United States). For nuclear localized proteins (BRD2 and NPM1), fluorescence intensity of the nuclear FITC signal was normalized to a background signal measurement proportionally sized to the nuclear area that was taken from the cytoplasm and subtracted from total fluorescence intensity prior to calculating intensity in arbitrary units. Total fluorescence intensity then was normalized to either the GV (for nuclear fluorescence) or oocyte (for cytoplasmic fluorescence) size and DNA signal intensity. The DNA signal intensity was normalized to background DAPI signal as described above for FITC signal normalization (Lee et al., 2018).

### Antibody Transfection Prior to *in vitro* Maturation (Experiments 2 and 3)

**FIGURE 1:**
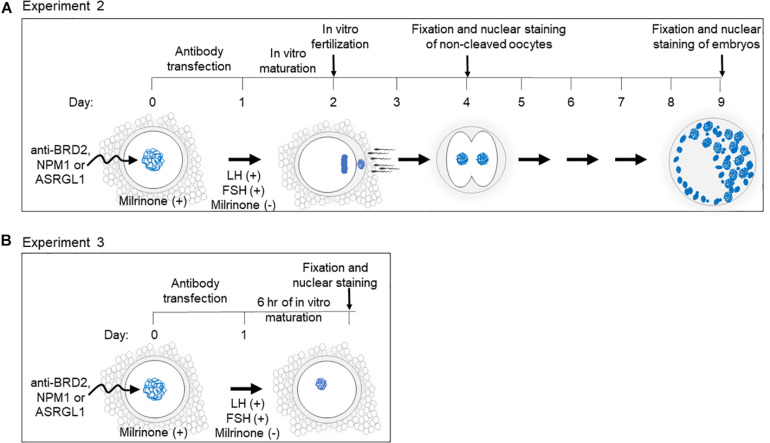
Schematic of experimental design and timeline for **(A)** Experiment 2 and **(B)** Experiment 3.

Protein inhibition for Experiments 2 and 3 (Figure 1) was performed by transfecting antibodies against candidate proteins into COCs as described by Li et al. (2015) using the Chariot peptide nanoparticle transfection reagent (Active motif, United States). This transfection method was previously verified by our group to reduce protein activity of PDE3A in cat oocytes (Li et al., 2015; Lee et al., 2018). PDE3A is a well-characterized phosphodiesterase required for cAMP degradation, which is necessary for meiotic resumption (Richard et al., 2001; Conti et al., 2002; Thomas et al., 2002). For the experiments in this study, we used PDE3A as a positive control for antibody transfection.

Antibody-Chariot complexes were assembled according to the manufacturer’s instructions by mixing 2 μL of Chariot in 50 μL of H_2_O with 1.5 μg of antibody in 50 μL of PBS for 30 min at room temperature. The assembled complexes were added to the COCs in equal amount of culture medium [MEM (Sigma-Aldrich, United States) supplemented with 1 mM pyruvate, 2 mM L-glutamine, 100 IU/mL penicillin, 100 μg/mL streptomycin and 4 mg/mL BSA]. COCs were incubated at 38.5°C with 5% CO_2_ for 3 h before adding another 200 μL of culture medium and further incubating for a total of 24 h (Figure 1). To prevent meiotic resumption during the transfection, 250 nM of milrinone (Sigma-Aldrich, United States) was added to the medium (except for in the fresh control treatment group) during the course of transfection (Grupen et al., 2006; Lee et al., 2018).

Following Chariot transfection, COCs were rinsed three times in SAGE blastocyst media (SAGE, Denmark) to remove milrinone. For IVM, COCs were incubated in a 50 μL drop of SAGE media containing 1 μg/mL ovine FSH (National Hormone and Pituitary Program, United States) and 1 μg/mL ovine LH (National Hormone and Pituitary Program, United States) for either 24 h (experiment 2) or 6 h (experiment 3).

#### Experiment 2: Effect of Protein Inhibition on *in vitro* Maturation, Fertilization, and Early Embryo Development

For each candidate protein, between 129 and 159 COCs in 2–4 replicates were divided amongst the following treatment groups: (1) mock transfection negative control (chariot transfection reagent alone), (2) PDE3A positive control (transfection with anti-PDE3A), (3) BRD2, NPM1 or ASRGL1 (candidate protein antibody transfection (Figure 1). Due to the inability to include fresh controls in every experimental repeat, the fresh control group could not be included in statistical analysis. However, it indicated the standard values in our regular experimental conditions.

Following antibody transfection described above and IVM, COCs were incubated for *in vitro* fertilization (IVF) with 2 × 10^6^ motile cells/mL of fresh or frozen-thawed domestic cat epididymal spermatozoa for 24 h. Oocytes then were mechanically denuded and gently rinsed three times in SAGE blastocyst medium before *in vitro* culture in 50 μL SAGE blastocyst media (5–15 presumptive zygotes per drop). Non-cleaved oocytes were removed from culture the following day, fixed in 4% PFA, and stained with DAPI (Figure 1). Oocyte meiotic stages were evaluated according to our standards (Comizzoli et al., 2011). Non-cleaved oocytes with fragmented or abnormal nuclei were classified as degraded. Cleaved embryos were culture for a total of 7 days (after IVF), fixed in 4% PFA, and stained with DAPI (Figure 1). Embryonic stage was determined by counting the number of blastomeres. Morula was classified as having 16–63 blastomeres and blastocysts were classified as having at least 64 blastomeres with the presence of a blastocele (Comizzoli et al., 2011).

#### Experiment 3: Antibody Transfection Followed by Short *in vitro* Maturation

A total of 148 oocytes (in 3 replicates) were equally and randomly allocated to one of the treatment groups (BRD2 inhibition, mock transfection; NMP1 inhibition, mock transfection). Oocytes were transfected with antibodies and underwent IVM as described above. After 6 h of IVM (Figure 1), oocytes were fixed in 4% PFA and processed for microtubule immunostaining (anti-beta-tubulin; Thermo MA5-11732; 1:150) as well as DAPI counterstaining following the protocol mentioned above.

### Statistical Analysis

Differences in fluorescence intensity in antibody staining of candidate proteins and 6 h IVM antibody transfection experiments were analyzed using a *T*-test. Antibody transfection experiments with multiple comparisons of oocytes and embryo stages were analyzed by ANOVA and Tukey’s multiple comparisons test (Prism v6.05; GraphPad Software, United States). Proportions of embryos were compared using a Chi-square test.

## Results

### Experiment 1: BRD2, NPM1, and ASRGL1 Protein Localization During Oocyte Development

**FIGURE 2:**
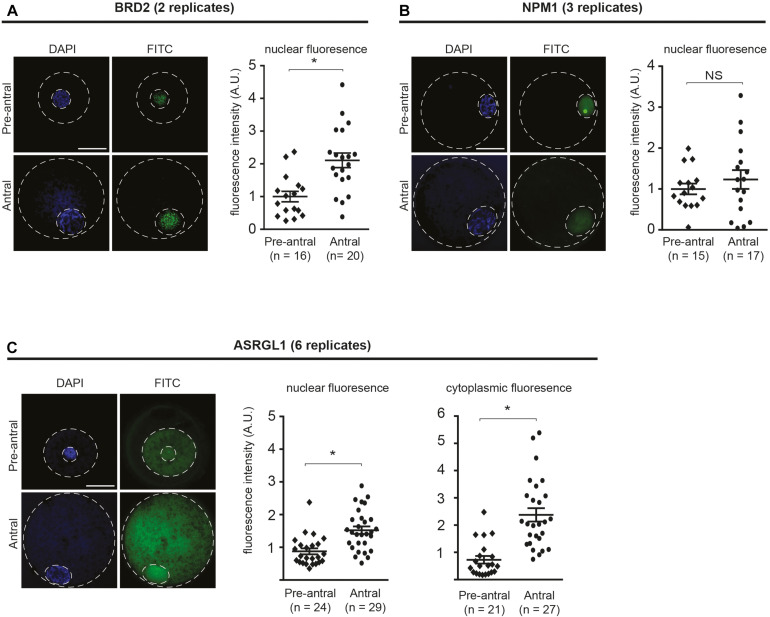
Localization of BRD2, NPM1, and ASRGL1 in pre-antral and antral oocytes. **(A)** BRD2, **(B)** NPM1, **(C)** ASRGL1. In left panels, representative images of pre-antral and antral oocytes. Outer dashed line delineates oocyte boundary and inner dashed line delineates germinal vesicle. Scale bars = 50 μm. In right panel graphs, quantification of normalized nuclear or cytoplasmic fluorescence intensity for each antibody in pre-antral and antral oocytes. Each data point indicates one oocyte. Bars show means ± SEM. *P < 0.05.

We first sought to compare the localization of candidate proteins in oocytes of pre-antral vs. antral follicular stage oocytes using immunofluorescent staining (Figure 2). Antibody specificity was confirmed by the presence of a protein band at the expected size using SDS-PAGE (Supplementary Figure 1). The two proteins involved in chromatin regulation, BRD2 and NPM1, were present in oocyte nuclei (Figures 2A,B). Specifically, BRD2 was detected in a punctate pattern throughout the nucleoplasm in both pre-antral and antral oocytes (Figure 2A). Quantification of fluorescence intensity revealed an increase of BRD2 protein (*P* < 0.05) in antral oocytes as compared to pre-antral counterparts (Figure 2A). NPM-1 was localized to the nucleus in both pre-antral and antral oocytes (Figure 2B). While there was no change in NPM-1 fluorescence intensity between the pre-antral and antral stages (*P* > 0.05), staining in pre-antral oocytes was concentrated in the nucleolus. Interestingly, in antral oocytes, NPM1 localization changed to a diffuse nucleoplasmic pattern (Figure 2B). The protein involved in cellular metabolism, ASRGL1 was present in a punctate pattern throughout the nucleoplasm and cytoplasm (Figure 2C). ASRGL1 fluorescence intensity increased (*P* < 0.05) in antral oocytes compared to pre-antral counterparts in both the nucleoplasm and the cytoplasm (Figure 2C). Changes observed in protein levels (BRD2 and ASRGL1) or subcellular localization (NPM1) suggested that these proteins might be involved in the critical transition period from an immature pre-antral to an antral oocyte (capable of meiotic maturation and competent for embryo development after fertilization).

### Experiment 2: Influence of Inhibition of BRD2, NPM1, and ASRGL1 on Subsequent Meiotic Resumption and Fertilization

To better characterize the role of candidate oocyte competence proteins, we inhibited BRD2, NPM1, and ASRGL1 in antral stage COCs by transfecting antibodies into COCs followed by IVM and IVF. We then examined the influence on subsequent meiotic resumption and early embryo development (Figure 1). To confirm transfection in each experiment, we included anti-PDE3A as a positive control. When BRD2 or NPM1 antibodies were transfected into COCs, a lower proportion of oocytes matured *in vitro*, were fertilized, and formed advanced embryos compared to mock transfection controls (*P* < 0.05) (Table 1). Specifically, only 15.2% of BRD2-inhibited and 1.2% of NPM1-inhibited oocytes were fertilized and cleaved as compared to 47.0 and 52.8% in respective mock transfection controls (Table 1). Analysis of chromatin status in oocytes that did not cleave revealed that majority of BRD2 and/or NPM1-inhibited oocytes were still arrested at the GV stage (46.9% for BRD2 and 72.2% for NPM1) or degraded (27.3% for BRD2 and 21.6% for NPM1) (Table 1). Percentages of oocytes reaching the metaphase II stage were lower after BRD2 inhibition (18.0%) and after NPM1 inhibition (1.2%) compared to the mock transfections (Table 1). While the majority of BRD2-, and NPM1-inhibited COCs failed to mature and be fertilized, a small number cleaved and were capable of forming embryos with less than 8 blastomeres (Table 1). Embryo development resulting from those two treatment groups was less successful than in mock transfection controls (Table 1). We suspected that these oocytes were inefficiently transfected with antibodies as we similarly observed a low level of cleavage and embryo development in PDE3A controls. Interestingly, transfection of ASRGL1 did not hinder (*P* > 0.05) maturation, fertilization, or embryo development compared to mock transfection controls (Table 1).

**TABLE 1:** Influence of the inhibition of BRD2, NPM1, and ASRGL1 in immature oocytes from antral follicles on the subsequent nuclear maturation and developmental competence.

Candidate protein (number of experimental replicates)	Treatment (total number of oocytes, total number of cleaved embryos across all replicates)	Percentages relative to the total number of oocytes					Proportions of embryo stages relative to the total number of cleaved embryos after 7 days of *in vitro* culture			
			
		Germinal vesicle oocytes*	Metaphase I oocytes*	Metaphase II oocytes*	Degraded*	Cleaved embryos	< 8-cells	8–16-cells	Morulae	Blastocysts
Fresh (4)	Fresh control (60, 38)	9.4 (± 4.7)	6.9 (± 5.5)	68.5 (± 6.0)	14.8 (± 5.0)	63.2 (± 2.8)	21.1	21.1	42.1	15.8
BRD2 (3)	Mock transfection (42, 22)	9.4^b^ (± 4.1)	2.5^a^ (± 2.5)	52.0^a^ (± 5.9)	36.2^a^ (± 5.1)	47.0^a^ (± 4.0)	41.0^a^	41.0	18.1	0.0
	PDE3A control (39, 2)	68.5^a^ (± 8.1)	1.9^a^ (± 1.9)	7.6^b^ (± 2.7)	22.0^a^ (± 6.5)	5.0^b^ (± 3.1)	100.0^a^	0.0	0.0	0.0
	BRD2 inhibition (50, 6)	46.9^ab^ (± 15.7)	7.9^a^ (± 5.1)	18.0^b^ (± 12.7)	27.3^a^ (± 9.3)	15.2^b^ (± 10.0)	100.0^a^	0.0	0.0	0.0
NPM1 (2)	Mock transfection (22, 13)	13.9^a^ (± 13.9)	12.5^a^ (± 12.5)	65.3^a^ (± 9.7)	8.3^a^ (± 5.6)	52.8^a^ (± 2.8)	23.1^a^	7.7^a^	69.2	0.0
	PDE3A control (23, 8)	55.9^ab^ (± 19.1)	2.7^a^ (± 2.7)	30.9^ab^ (± 5.9)	10.5^a^ (± 7.0)	28.3^b^ (± 3.3)	87.5^a^	12.5^a^	0.0	0.0
	NPM-1 inhibition (53, 1)	72.2^b^ (± 2.2)	5.0^a^ (± 5.0)	1.2^b^ (± 1.2)	21.6^a^ (± 8.2)	1.2^c^ (± 1.2)	100.0^a^	0.0	0.0	0.0
ASRGL1 (3)	Mock transfection (34, 15)	24.7^b^ (± 9.8)	14.1^a^ (± 10.0)	51.3^ab^ (± 9.2)	9.8^a^ (± 5.3)	44.2^ab^ (± 9.7)	40.0^a^	0.0	46.7^a^	13.3^a^
	PDE3A control (32, 2)	72.7^a^ (± 2.7)	0.0^a^ (± 0.0)	13.7^b^ (± 8.3)	13.7^a^ (± 6.9)	9.5^b^ (± 9.5)	100.0^a^	0.0	0.0	0.0
	ASRGL1 inhibition (76, 42)	13.9^b^ (± 6.1)	6.2^a^ (± 3.6)	68.9^a^ (± 9.6)	11.1^a^ (± 7.4)	51.0^a^ (± 4.5)	26.2^a^	14.3	45.2^a^	14.3^a^

*Nuclear status from non-cleaved oocytes; Metaphase II values also include oocytes that were fertilized and cleaved. Values expressed as mean ± standard error of the mean (SEM), except for embryo stages (overall percentages). Within the column of a given protein, percentages with different superscripts a–c differ (P < 0.05). Fresh controls were performed on separate batches of oocytes and thus not included in the statistical analysis.

### Experiment 3: Influence of Inhibition of BRD2 and NPM1 on Early Meiotic Maturation Events

Experiments assessing embryo development showed that BRD2 and NPM1-inhibited COCs were incapable of fertilization and suggested this failure may be due to failed meiotic maturation. To precisely determine the stage at which BRD2 or NPM1 were critical to maturation, we analyzed inhibited COCs during the time at which they were just beginning to respond to the hormonal maturation signals LH and FSH (6 h of IVM).

**FIGURE 3:**
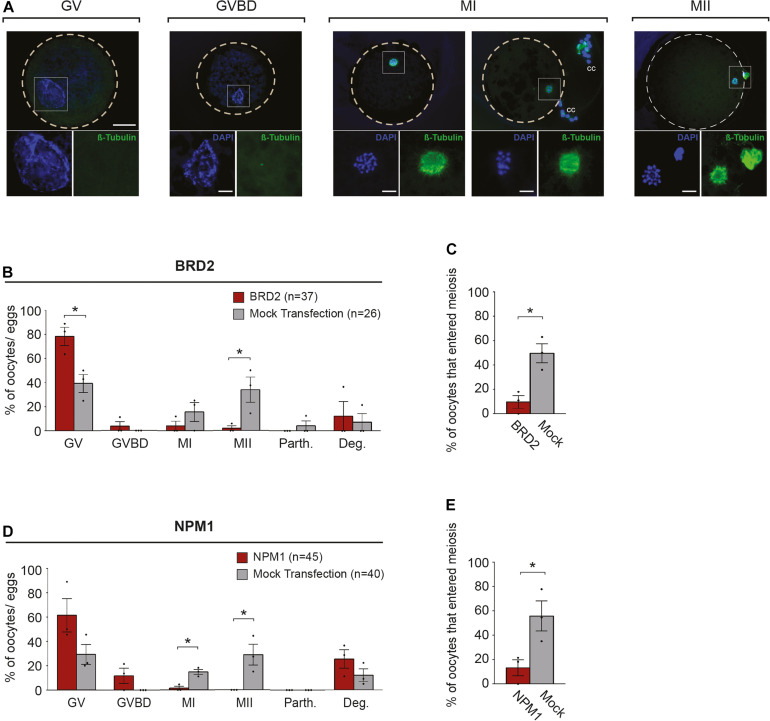
Meiotic progression of BRD2 and NPM1-inhibited oocytes following 6 h of *in vitro* maturation (IVM). **(A)** Representative images of meiotic stages: GV- germinal vesicle, GVBD- germinal vesicle break-down, MI- metaphase I, and MII- metaphase II. Top images: merged FITC (anti-β-Tubulin) and DAPI images. Outer dashed line delineates oocyte boundary. Scale bar = 50 μm. Lower images in squares: magnified area of chromatin configuration (DAPI) and meiotic spindle (β-Tubulin). Scale bar = 10 μm. cc = cumulus cells. **(B,D)** Bar graphs showing the percentage of oocytes in each meiotic stage following protein inhibition and 6 h of IVM. GV, GVBD, MI, MII, Parth.- parthenotes, Deg.- degraded. **(C,E)** Bar graphs showing percentage of oocytes that entered meiosis following 6 h of IVM. Red bars = indicated protein inhibited, Gray bars = mock transfection control. Bars show mean ± SEM (3 replicates). *P < 0.05.

**FIGURE 4:**
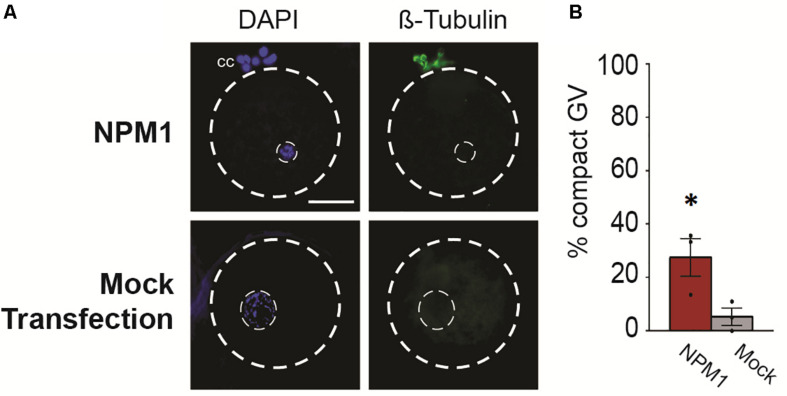
Compact nuclear phenotype of NPM1-inhibited oocytes. **(A)** Representative images of NPM1-inhibited oocytes with compact germinal vesicle (GV) phenotype. Meiotic spindle immunostained with anti-β-Tubulin, DNA stained with DAPI. Outer dashed line delineates oocyte boundary, inner dashed line delineates germinal vesicle. Scale bar = 50 μm. **(B)** Bar graph shows% of oocytes with a compact GV phenotype following 6 h IVM. Bars show mean ± SEM (3 replicates). *P < 0.05.

Following 6 h of exposure to IVM conditions, we analyzed the nuclear stage by observing chromatin configuration and the meiotic spindle using anti-β-Tubulin (Figure 3A). We found that BRD2-inhibited oocytes remained at the GV stage (*P* < 0.05) whereas mock transfection controls entered meiosis in higher proportions (*P* < 0.05) (Figures 3B,C). Similarly, only a very small percentages of NPM1-inhibited oocytes could resume meiosis compared to controls (*P* < 0.05) (Figures 3D,E). In addition, NPM1 inhibition caused a phenotype—GV compaction—that was not observed with BRD2 inhibition (Figure 4A). As compared to mock controls, GV compaction occurred in 27% of NPM1-inhibited oocytes compared to only 9% of mock transfection control oocytes (*P* < 0.05) (Figure 4B). While it is unlikely meiosis could proceed through to the metaphase II stage in just 6 h, it is likely that a small proportion of control COCs escaped milrinone inhibition and entered meiosis during the 1 day transfection period. These experiments showed that BRD2 and NPM1 were important for early meiotic maturation events as inhibition of either of the two resulted in oocytes that fail to respond to exogenous LH and FSH meiotic maturation signals.

## Discussion

Our results identified two novel regulators of oocyte meiotic competence, the nuclear transcription factor BRD2 and the nucleolar protein NPM1. Using immunostaining, we observed changes in protein levels and/or localization for all three proteins, suggesting they are regulated during development, and may contribute to fertility. Using protein inhibition assays, we targeted candidate proteins by transfecting antibodies against candidate proteins into COCs. We found that inhibition of both nuclear proteins, BRD2 and NPM1, resulted in oocytes that could not mature, be fertilized nor form embryos. ASRGL1 inhibition did not hinder meiotic maturation or embryo development. Further analysis of early meiotic resumption stages by observation of the meiotic spindle and chromatin configuration demonstrated that inhibition of BRD2 and NPM1 renders oocytes unable to resume meiosis and thus unable to participate in fertilization and embryo development. Together, our data show that we have identified BRD2 and NPM1 as two novel regulators of oocyte meiotic competence.

By immunostaining pre-antral and antral oocytes, we observed BRD2 to be present in the nucleus during folliculogenesis. BRD2 is known to be involved in chromatin remodeling and transcriptional regulation (Trousdale and Wolgemuth, 2004; Peng et al., 2006; Hnilicová et al., 2013). As expected, we observed BRD2 in the nucleus, similar to the nuclear localization of BRD2 previously reported in various cell types including in developing mouse oocytes (Trousdale and Wolgemuth, 2004). Quantification of fluorescence intensity of BRD2 showed a slight upregulation of BRD2 protein from the pre-antral to the antral stage. While this result differs from that our previous proteomics study in which BRD2 was found to be downregulated in antral oocytes as compared to pre-antral, we suspect that differences between the studies could be attributed a low level of somatic cell contamination in the proteomics study (Lee et al., 2018).

Our experiments showed that BRD2 played a role during meiotic resumption as inhibition of BRD2 in antral stage COCs prevented meiotic maturation and oocytes remained at the GV stage despite the presence of exogenous LH and FSH meiotic maturation signals. In the only former study on BRD2 during folliculogenesis (Trousdale and Wolgemuth, 2004), demonstrated that BRD2 expression was highly regulated throughout mouse oocyte development and predicted that BRD2 could play a role in meiotic resumption as the yeast homolog has been shown to be critical during meiosis (Chua and Roeder, 1995; Kanatsu-Shinohara et al., 2000; Filippakopoulos et al., 2012). While it has never formally been tested, here we found that in the cat, BRD2 is indeed important for meiotic resumption. We hypothesize that disrupting BRD2 in antral oocytes, a stage during which transcription is largely quiescent, causes aberrant transcription that is detrimental to proper meiotic resumption. Indeed, BRD2 has been shown to regulate the transcription of critical cell cycle genes via binding to acetylated histone 4 (Owen et al., 2000; Sinha et al., 2005).

In our second set of experiments, where we analyzed very early stages of meiotic resumption, most BRD2-inhibited oocytes did not show any sign of resuming meiosis while control oocytes had begun to respond to LH and FSH signals. In fact, BRD2-inhibited oocytes appeared to be normal, healthy GV stage cells, indicating that the requirement for BRD2 is early, perhaps when cell cycle checkpoints for meiotic resumption are critical. Our analysis of BRD2, together with the existing studies of BRD2 molecular mechanisms during mitosis, meiosis and transcriptional regulation, leads us to predict that in our experiments, cell cycle regulation is disrupted thereby preventing early meiotic events from initiating. Although we cannot rule out the notion that BRD2 in cumulus cells contributes to the observed phenotype, former experiments in the mouse show that BRD2 localization is not disrupted in FSH or GDF9 mutants (Trousdale and Wolgemuth, 2004). This suggests that the effect is likely driven by BRD2 in the oocyte itself rather than in cumulus cells that signal oocytes to mature through their FSH and LH receptors (Buccione et al., 1990). While many studies have demonstrated BRD2 is critical during mitosis and even meiosis in sperm cells, our work shows for the first time its role during meiotic maturation in the oocyte in a large, non-rodent mammalian system (Philipps et al., 2008). In future experiments, it would be interesting to determine if BRD2 in cat oocytes is also regulating cell cycle by regulating acetylated histone 4 as has been shown in other systems (Gursoy-Yuzugullu et al., 2017).

NPM1 is a nuclear protein involved in chromatin regulation, RNA transport and ribosome biogenesis (Box et al., 2016). During cat folliculogenesis, we observed NPM1 protein localization change from nucleolar localization in pre-antral stage oocytes to diffuse throughout the nucleoplasm in antral oocytes. This nucleolar to nucleoplasmic localization pattern of NPM1 localization has also been reported in cell lines (Korgaonkar et al., 2005). That study found that a change from nucleolar to nucleoplasmic localization was induced by stressors and was followed by transcription of genes involved in cell-cycle arrest, DNA repair and apoptosis. Intriguingly, the closely related NPM2 protein, which is in the same nucleophosmin family as NPM1, is known to be important for meiotic maturation. NPM2 null mutant female mice are infertile or subfertile due to aberrant nucleolar chromatin organization that leads to early embryonic lethality (Burns et al., 2003). In particular, NPM2 is required for the surrounded nucleolar (SN) chromatin configuration that is associated with the ability to resume meiosis in many systems including mice. While the cat differs in that it does not acquire the SN chromatin configuration as a part of normal folliculogenesis, NPM1 structure is very similar to NPM2, with the exception of also possessing a nucleolar localization signal that is not present in NPM2 (Burns et al., 2003; Fuente et al., 2004; Comizzoli et al., 2011; Box et al., 2016). We postulate that NPM1, like NPM2, is important for proper chromatin organization required for meiotic resumption. Furthermore, by examining early meiotic maturation in NPM1-inhibited oocytes, we found a significant proportion of GVs had become compacted, perhaps indicating that some chromatin remodeling events required for meiosis are beginning to occur or are misregulated and thus insufficient to proceed with normal chromosome segregation and produce fertilizable eggs. Future investigation of NPM1 in the cat should explore how NPM1 interacts with known nucleophosmin-interacting pathways and why misregulation can lead to the nuclear compaction phenotype we observed.

ASRGL1 was present in the nucleoplasm as well as the cytoplasm, a localization pattern similar to that of ASRGL1 in other somatic cell types and in testicular tissue (Bush et al., 2002; Dieterich et al., 2003; Huvila et al., 2018). While we did observe an increase in both nuclear and cytoplasmic ASRGL1 at the antral stage, inhibition of ASRGL1 did not interfere with meiotic maturation or embryo development. In fact, ASRGL1-inhibited COCs tended to have higher blastocyst rates as compared to mock transfection controls. Although the trend was not statistically significant, it was an intriguing observation. Although we did not observe any significant phenotypes following ASRGL1 inhibition, we cannot rule out the possibility that our ASRGL1 antibody inefficiently inhibits the protein in COCs or that a higher concentration, or longer inhibition may reveal a role for ASRGL1.

Together, the protein localization analysis adds to our previous proteomic study (Lee et al., 2018) by demonstrating that these proteins are present in developing cat oocytes and that their abundance and/or localization is regulated. Our investigation revealed that two of the three candidate proteins, BRD2 and NPM1, are required for meiotic competence. Although the mechanisms by which BRD2 and NPM1 contribute to competence remain unknown in this system, previous work in other animals provides several potential hypotheses to be tested in future studies. This could be studied by analyzing proteins computationally predicted to interact with BRD2 and NPM1 or shown in other model systems to interact with them. Furthermore, future analysis could examine more general markers of oocyte competence and health such as apoptotic markers or by analyzing reactive oxygen species that can be damaging to overall oocyte viability.

In summary, this study highlights the fact that the molecular underpinnings of gametes are often similar to those found in somatic cells and are adapted for the specialized cell divisions, regulation of genomic DNA configuration and cellular metabolism required by gametes during meiosis. While the proteins we investigated have been studied in other somatic cells, we show here for the first time that BRD2 and NPM1 are critical regulators of meiotic maturation in a large, non-inbred mammalian system. The discovery that these proteins are important during oocyte development in the domestic cat will assist in developing novel fertility preservation techniques focusing on oocyte preservation and IVM. Not only will these techniques contribute to endangered felid conservation but also hold promise for use in human reproductive medicine.

## Data Availability Statement

The raw data supporting the conclusions of this article will be made available by the authors, without undue reservation.

## Author Contributions

DC and P-CL designed the experiments. DC performed the experiments, analyzed the data, and wrote the manuscript. PC contributed to study design, data interpretation, and article revision. All authors contributed to the article and approved the submitted version.

## Conflict of Interest

The authors declare that the research was conducted in the absence of any commercial or financial relationships that could be construed as a potential conflict of interest.

## Supplementary Material

Supplementary Figure 1 Western blots of candidate proteins with respective IgG negative control antibodies. **(A)** BRD2, **(B)** NPM1, **(C)** ASRGL1.

Supplementary Figure 2 Representative images of rabbit or mouse non-immune IgG negative controls for BRD2, NPM1, and ASRGL1. Scale bars = 50 μm.
