# Supplementary figures and images for: Oocyte Meiotic Competence in the Domestic Cat Model: Novel Roles for Nuclear Proteins BRD2 and NPM1
Source: Front Cell Dev Biol. 2021 May 3;9:670021. doi: 10.3389/fcell.2021.670021 (PMC8126674; doi:10.3389/fcell.2021.670021)

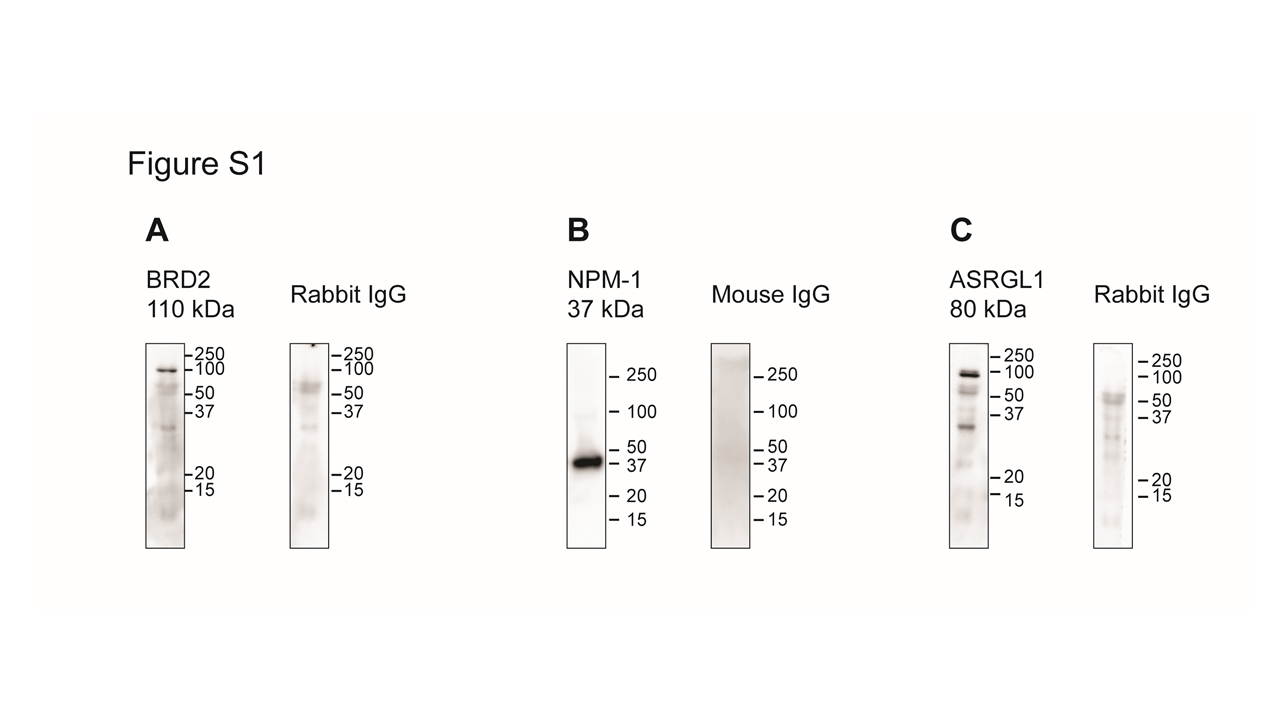

Supplement: Supplementary Figure 1 — Western blots of candidate proteins with respective IgG negative control antibodies. (A) BRD2, (B) NPM1, (C) ASRGL1. [file Image_1.TIF]

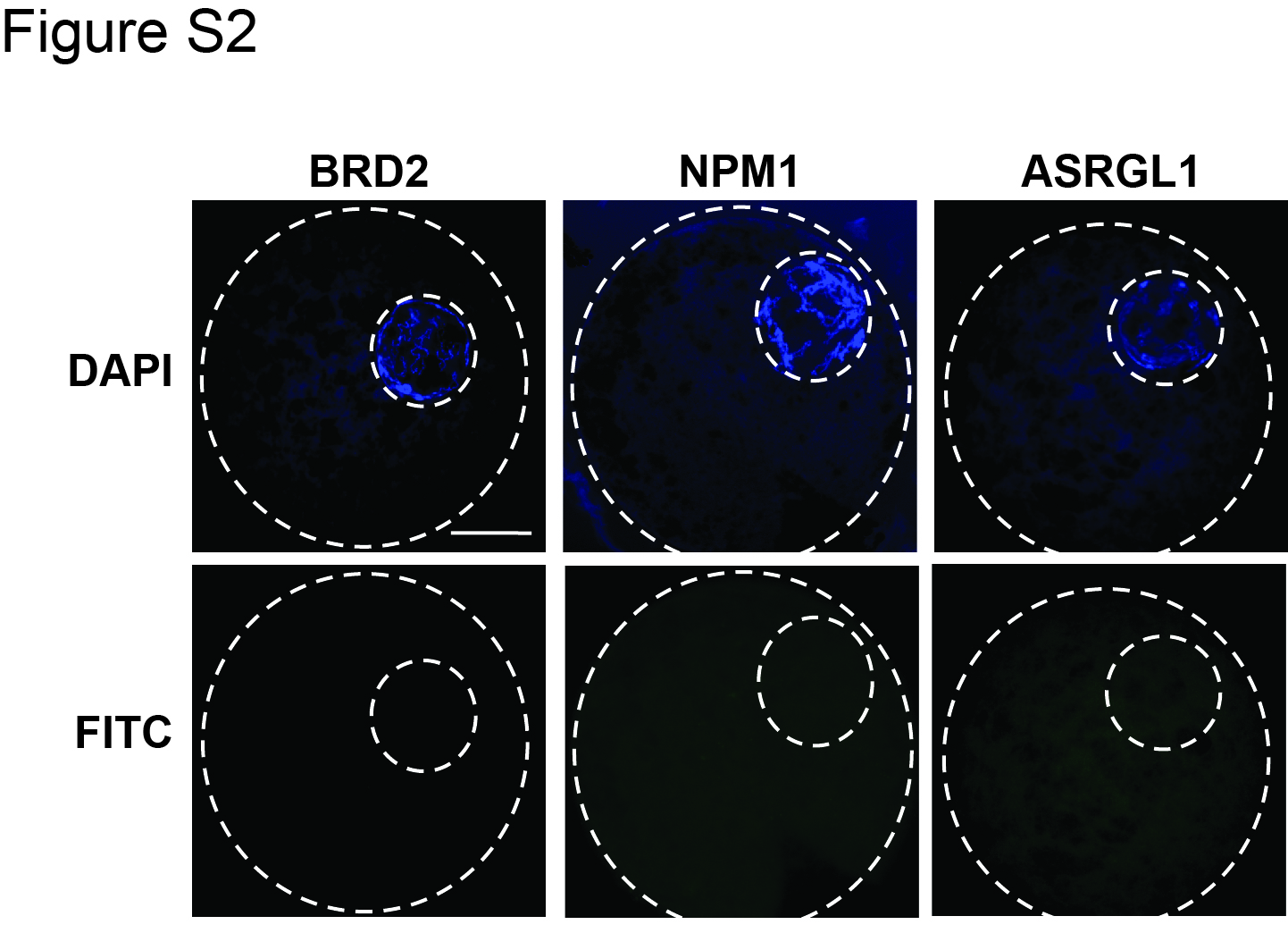

Supplement: Supplementary Figure 2 — Representative images of rabbit or mouse non-immune IgG negative controls for BRD2, NPM1, and ASRGL1. Scale bars = 50 μm. [file Image_2.TIFF]
